# Performance-Enhanced Piezoelectric Micromachined Ultrasonic Transducers by PDMS Acoustic Lens Design

**DOI:** 10.3390/mi15060795

**Published:** 2024-06-17

**Authors:** Licheng Jia, Yong Liang, Fansheng Meng, Guojun Zhang, Renxin Wang, Changde He, Yuhua Yang, Jiangong Cui, Wendong Zhang, Guoqiang Wu

**Affiliations:** 1 Key Laboratory of Instrumentation Science and Dynamic Measurement, North University of China, Taiyuan 030051, China; s202106112@st.nuc.edu.cn (Y.L.); sz202206139@st.nuc.edu.cn (F.M.); zhangguojun1977@nuc.edu.cn (G.Z.); wangrenxin@nuc.edu.cn (R.W.); hechangde@nuc.edu.cn (C.H.); 13513641974@163.com (Y.Y.); cuijiangong99999@163.com (J.C.); wdzhang@nuc.edu.cn (W.Z.); 2 Hubei Key Laboratory of Electronic Manufacturing and Packaging Integration, Institute of Technological Sciences and the School of Microelectronics, Wuhan University, Wuhan 430072, China; wuguoqiang@whu.edu.cn

**Keywords:** PMUT array, PDMS, acoustic lenses, sensitivity

## Abstract

This paper delves into enhancing the performance of ScAlN-based Piezoelectric Micromachined Ultrasonic Transducers (PMUTs) through the implementation of Polydimethylsiloxane (PDMS) acoustic lenses. The PMUT, encapsulated in PDMS, underwent thorough characterization through the utilization of an industry-standard hydrophone calibration instrument. The experimental results showed that the ScAlN-based PMUT with the PDMS lenses achieved an impressive sensitivity of −160 dB (re: 1 V/μPa), an improvement of more than 8 dB compared to the PMUT with an equivalent PDMS film. There was a noticeable improvement in the −3 dB main lobe width within the frequency response when comparing the PMUT with PDMS encapsulation, both with and without lenses. The successful fabrication of high-performance PDMS lenses proved instrumental in significantly boosting the sensitivity of the PMUT. Comprehensive performance evaluations underscored that the designed PMUT in this investigation surpassed its counterparts reported in the literature and commercially available transducers. This encouraging outcome emphasizes its substantial potential for commercial applications.

## 1. Introduction

The field of underwater acoustics continually strives for advancements in sensor technologies to meet the demands of diverse applications, such as environmental monitoring, marine research, and underwater communication systems [1,2,3,4,5,6]. In the field of underwater acoustics, ultrasonic transducers play a crucial role. Ultrasonic transducers exhibit exceptional performance in underwater environments, providing essential support for various key applications. Whether in underwater communication systems, marine research, or environmental monitoring, the role of ultrasonic transducers is paramount. They have the ability to convert acoustic signals into electrical signals, facilitating the transmission, reception, and interpretation of these signals underwater.

To date, the most advanced transducer available in the market are constructed using bulky piezoceramic materials through traditional precision manufacturing technologies [7,8,9,10]. However, the emergence of microelectromechanical system (MEMS) technology has sparked interest in aluminum nitride (AlN)-based PMUTs due to their compatibility with CMOS processes [11,12,13]. In comparison to other ultrasonic transducer technologies, AlN-based PMUTs often require lower bias voltages, enhancing their energy efficiency [14,15]. The ease of fabrication and compatibility with mainstream system-in-packaging (SiP) technologies further solidify PMUTs as a promising choice for next-generation ultrasonic applications [16,17,18]. The sensitivity and directivity of AlN-based PMUTs are paramount for accurately detecting and analyzing underwater sounds, ranging from marine life communication to detecting potential threats.

One way to enhance the performance of AlN-based PMUTs is improving the piezoelectric coefficient of the materials and innovative structure designs, such as inducing a change in the mode shape from Gaussian-like to piston-like [19,20,21]. Other approaches to enhance AlN-based performance include manipulating the material properties through the utilization of dimpled piezoelectric elements, implementing a dual-electrode bimorph design [22], and adopting a dual-electrode design [23]. Methods of sound focusing involve controlling the propagation and concentration of sound waves to achieve precise manipulation of specific areas. Common techniques for sound focusing include acoustic lense focusing and phased array technology. In recent years, the integration of acoustic lens technology has emerged as a promising avenue for advancing transducer capabilities. Acoustic lenses, drawing inspiration from their optical counterparts, offer the potential to focus, steer, and enhance acoustic signals. This technology presents a transformative opportunity to elevate PMUT sensitivity, improve noise resolution, and extend detection ranges. PDMS, known for its biocompatibility, flexibility, and ease of fabrication, presents itself as an ideal material for constructing acoustic lenses tailored to PMUTs. Acoustic lenses play a pivotal role in focusing and directing incoming acoustic waves onto PMUTs, optimizing sensitivity, and improving the overall functionality of the transducer.

This paper describes the innovative design and implementation of PDMS acoustic lenses to improve the performance of ScAlN-based PMUTs in underwater environments. The underwater domain presents unique challenges for PMUTs, requiring specialized enhancements to overcome sensitivity and directivity issues. By focusing on the incorporation of PDMS acoustic lenses, this study aims to address these challenges and open up new possibilities for PMUT applications in underwater scenarios.

## 2. PMUT Array Design

**Figure 1:**
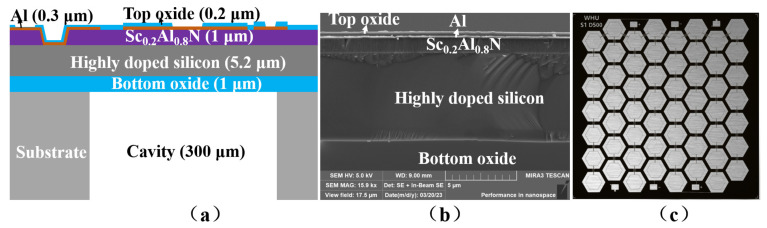
ScAlN-thin-film-based PMUT. (**a**) Cross-sectional view of the PMUT structure. (**b**) Cross-sectional SEM image of a deposited ScAlN thin-film. (**c**) Optical microscope image of a fabricated PMUT.

The cross-sectional view of the discussed PMUT, crafted using a piezoelectric-on-cavity silicon-on-insulator (CSOI) platform, is showcased in Figure 1a. It comprises a 1 μm-thick ScAlN piezoelectric thin film, positioned between a 0.15 μm thick molybdenum layer serving as the top electrode and a 5.2 μm thick highly doped silicon (HDS) device layer acting as the bottom electrode. The cross-sectional SEM image of the sputtered ScAlN piezoelectric thin-film is illustrated in Figure 1b. Figure 1c shows an optical microscope image of the PMUT. The PMUT, with dimensions of 4 mm × 4 mm, is configured in a honeycomb architecture, as depicted in Figure 1c. The key design parameters of the reported PMUT array are listed in Table 1.

## 3. Lens Design

**Figure 2:**
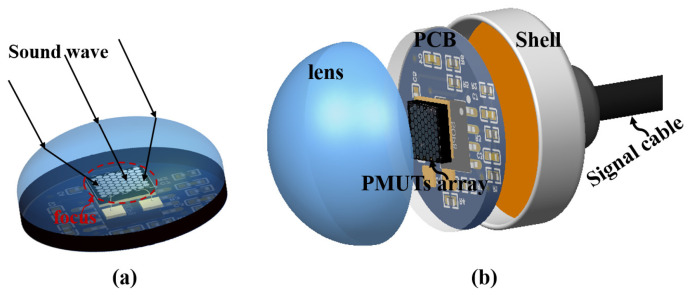
The acoustic lenses’ structure. (**a**) The acoustic lenses’ focusing process. (**b**) The packaging structure of the PMUT acoustic lenses.

As acoustic waves propagate, the lens converges or diverges them to achieve a desired focal point. This focusing mechanism enhances the sensitivity and resolution of PMUTs, enabling precise detection and characterization of targets in the acoustic field. The effectiveness of the acoustic lens in shaping and concentrating sound waves contributes to improved performance in applications such as medical imaging, underwater communication, and industrial sensing. The process of acoustic lens focusing involves the precise manipulation of sound waves through a carefully designed lens structure. The acoustic lens-focusing process is shown in Figure 2a. Figure 2b illustrates the packaging structure of the PMUT acoustic lens, encompassing the PDMS acoustic convex lens, PMUT, preamplifier circuit, and packaging tube.

The amplitude of the transmitted wave is subject to variations induced by both the attenuation occurring within the lens and the refraction at its boundary. These factors play a crucial role in shaping the characteristics of the transmitted wave, impacting its overall behavior and signal integrity. Understanding and managing these effects is essential for optimizing the performance of the acoustic lens and ensuring accurate and reliable signal transmission in applications such as underwater communication systems.

The transmission coefficient, $T_{l}$, through the lens material can be articulated as:

(1) $$T_{l}=10^{-\alpha (f)*H/10}$$

where

(2) $$\alpha \left(f\right)=\alpha \left(0\right)+\alpha_{f}*\left(f-f_{0}\right)$$

where H and *f* are the thickness of the material and the frequency, respectively. α(f) is the frequency-dependent attenuation coefficient of the material. The attenuation coefficient of the material at reference frequency $f_{0}$ is represented by α(0).

It is essential to emphasize that $T_{l}$ exponentially depends on frequency, influencing the shape of the frequency impulse response. This leads to a shift in the center frequency and a reduction in bandwidth. The attenuation coefficient is particularly crucial for high-frequency transducers, as signals at these frequencies can be completely attenuated.

The transmission coefficient ($T_{r}$) for transmitted power after partial refraction at the boundary is given by:

(3) $$T_{r}=\frac{4Z_{l}Z_{m}}{{\left(Z_{m}+Z_{l}\right)}^{2}}$$

where $Z_{m}$ and $Z_{l}$ are the acoustic impedance of the imaging medium and lens material, respectively. The transmitted wave power increases when the ratio $Z_{m}$/$Z_{l}$ is close to 1, which is a constraint on the density and speed of sound in the selected material.

The total transmitted power ($T_{tot}$) is then given by the product of the two transmission coefficients:

(4) $$T_{tot}=T_{l}*T_{r}$$

To optimize the transmitted wave amplitude, Equation (6) suggests that the lens should be minimized in thickness and the material should possess a low attenuation coefficient. In general, the formula commonly used to determine the required radius of curvature (Rc) for an acoustic lens is:

(5) $$R_{C}=F_{eff}\times \frac{V_{H}-V_{L}}{V_{L}}$$

where $F_{eff}$ is the desired effective geometric focal distance, $V_{H}$ is the sound speed of human tissue, and $V_{L}$ is the sound speed of the acoustic lens material ($V_{L}$ < $V_{H}$ in this study).

Furthermore, the thickness of the acoustic lens (TL) is expressed by the following equation:

(6) $$T_{L}=R_{C}-\sqrt{R_{C}^{2}-{\left(D/2\right)}^{2}}$$

where $R_{C}$ is the radius of curvature and *D* is the aperture length. The characteristics of the acoustic lens can be summarized in two aspects. Firstly, the radius of curvature of the acoustic lens increases as the sound speed of the acoustic lens decreases. Secondly, the thickness of the acoustic lens decreases as the radius of the lens increases, considering the same aperture size.

Achieving a suitable impedance match between the acoustic lens and the working environment medium is imperative to prevent the occurrence of imaging artifacts. This meticulous alignment ensures optimal signal transmission and reception, contributing to the overall quality and accuracy of the imaging process. Undoubtedly, if the wave undergoes substantial reflection within the lens, it leads to the generation of secondary echoes, appearing as reverberations in the ultrasound image. To mitigate this phenomenon, minimizing the power ratio of the reflected to transmitted wave (υ) is crucial.

(7) $$\upsilon =\frac{{\left(Z_{m}-Z_{l}\right)}^{2}}{4Z_{m}Z_{l}}$$

**Figure 3:**
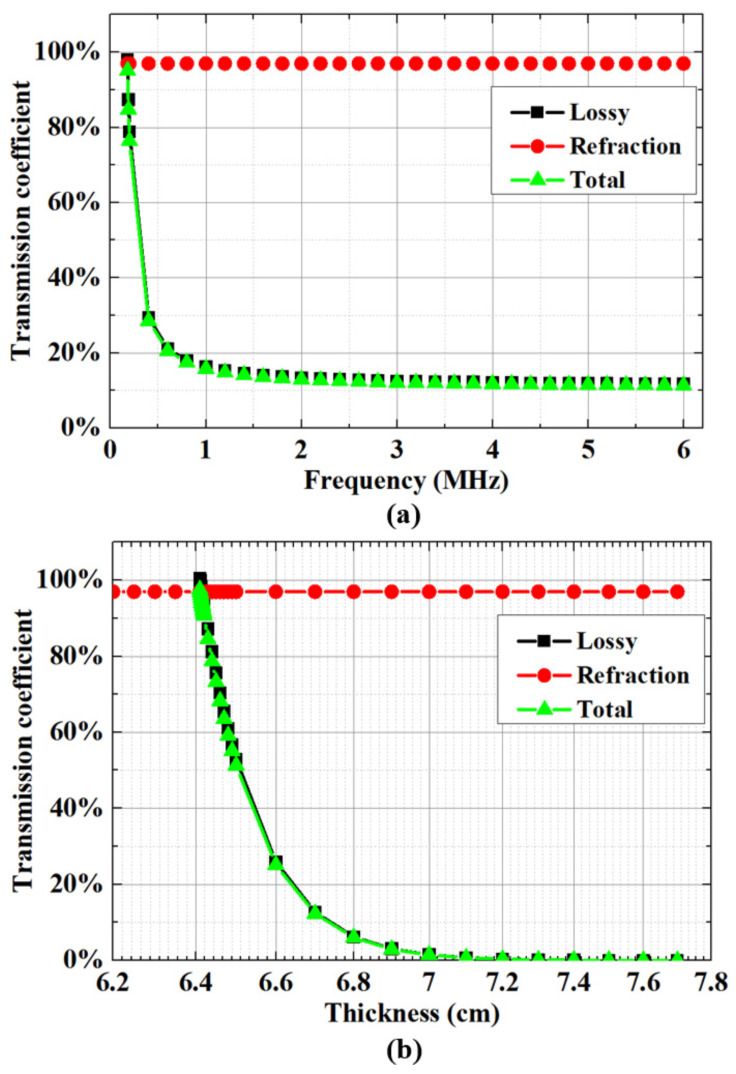
Relationship among frequency, thickness, and transmission coefficient of acoustic lenses. (**a**) Calculated transmission coefficients at various frequencies for an acoustic lens thickness of 5 mm. (**b**) Calculated transmission coefficients at 220 kHz for various thicknesses.

The optimal acoustic transmission and minimal impedance mismatches in ultrasound imaging are influenced by specific parameters, especially the impedance matching between the lens material and imaging medium. Figure 3 visually demonstrates the interplay among frequency, thickness, and the transmission coefficient of the acoustic lens. Additionally, Figure 3a outlines the critical relationship between the transmission coefficient and frequencies. High-frequency transducers, crucial for ultrasound imaging, are significantly impacted by the attenuation coefficient, which can potentially completely dampen signals at these frequencies. Moreover, the thickness of the acoustic lens plays a substantial role in reducing both total acoustic attenuation and the acoustic attenuation of the lens material, as illustrated in Figure 3b.

## 4. Lens Fabrication

**Figure 4:**
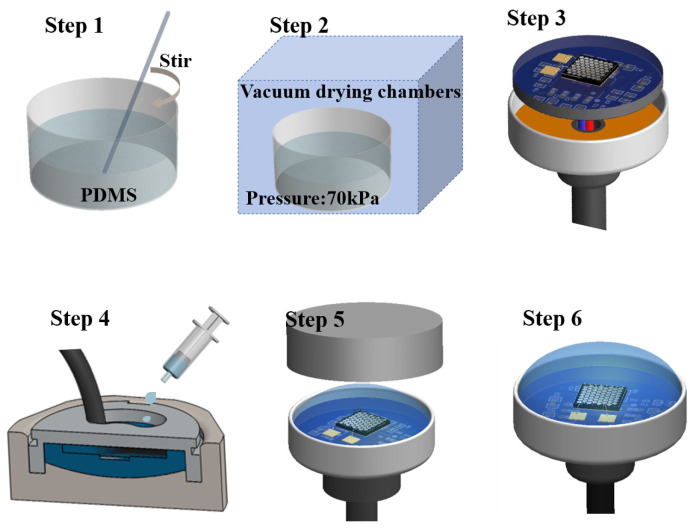
The PMUT acoustic lenses’ fabrication process.

The stepwise process for encapsulating the PMUT with an acoustic lens is depicted in Figure 4. Initially, the required amount of PDMS is measured and thoroughly mixed in a beaker to eliminate any entrapped air bubbles (step 1). Subsequently, the PDMS is subjected to degassing in a vacuum drying chamber oven (step 2), with steps 1 and 2 repeated until complete removal of the bubbles. Moving forward, the PMUT and preamplifier circuitry are assembled within a custom packaging shell (step 3). The PDMS is carefully poured into the tube shell, positioned in the oven, and heated at 90 °C for 120 min (step 4). Following this, the mold is removed, allowing the material to cool, and the solidified PMUT acoustic lens is released from the cavity (step 5). The accomplished PMUT acoustic lens is visually presented in step 6.

PDMS lenses with a carefully selected radius of curvature have been successfully manufactured. Full details of these lenses can be found in Table 2.

## 5. Performance Characterization

**Figure 5:**
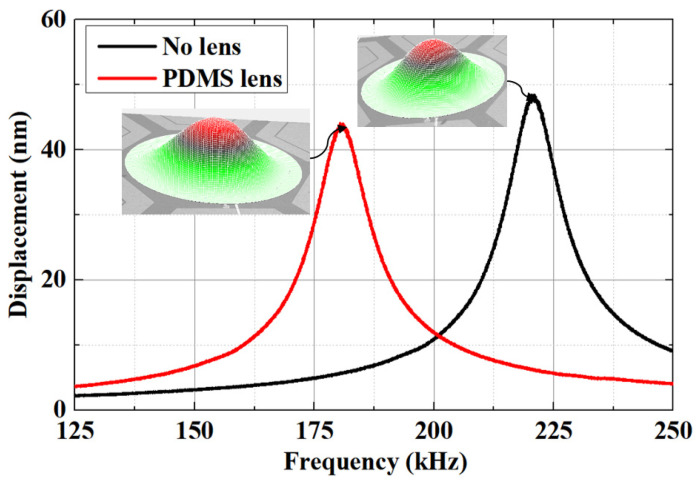
Comparison frequency responses of the PMUT with no lenses and PDMS lenses, obtained by a Polytec MSA-600 LDV.

Utilizing the advanced capabilities of the PolyTec MSA-600 LDV ensures accurate characterization, facilitating a comprehensive analysis of the PMUT’s frequency response. This approach is essential to evaluate and optimize the performance of the PMUT for its intended applications. Figure 5 shows a comparison of the frequency responses of the PMUT without a lens and with a PDMS lens, obtained using a Polytec MSA-600 LDV. The resonant frequencies of the PMUT are measured at 221 kHz without a lens and 183 kHz with the PDMS lens.

**Figure 6:**
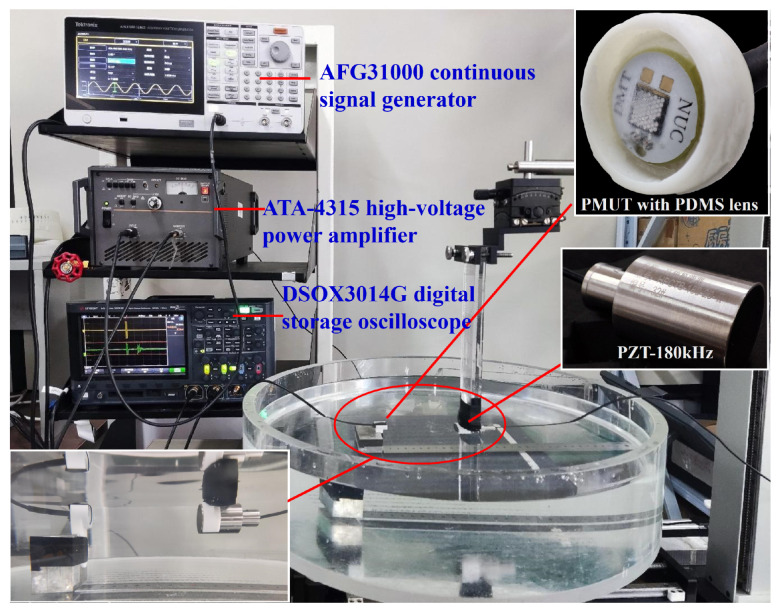
The PMUT sensitivity test diagram with PDMS lens (step 2).

The measurement of acoustic pressure sensitivity comprises two steps. As depicted in Figure 6, initially, a pair of standard piezoelectric transducers with a resonance frequency of 180 kHz (this transducer is made from piezoelectric ceramic wafers and is a type HPCTB-180-20-II standard piezoelectric transducer, calibrated and certified by the first-level metrological station for underwater acoustics of China’s defense science and technology industry) is fixed at the transmitting and receiving ends, facing each other with a distance of 10 cm. The transmitting end is driven by an AFG31000 continuous signal generator, powering an ATA-4315 high-voltage power amplifier to apply a 30 Vpp sinusoidal pulse AC signal with five cycles from 100 kHz to 300 kHz. The voltage amplitude at the receiving end is captured using a DSOX3014G digital storage oscilloscope, which is then converted to determine the acoustic pressure (P) on the surface of the standard piezoelectric transducer (PZT-180 kHz). Subsequently, while maintaining the transmitting end and its parameter settings unchanged, the standard piezoelectric transducer (PZT-180 kHz) at the receiving end is replaced with the PMUT equipped with a PDMS lens/film. The voltage signal amplitude (U) at the receiving end is then re-collected, and the acoustic pressure sensitivity of the PMUT with the PDMS lens/film is calculated using the values of P and U.

**Figure 7:**
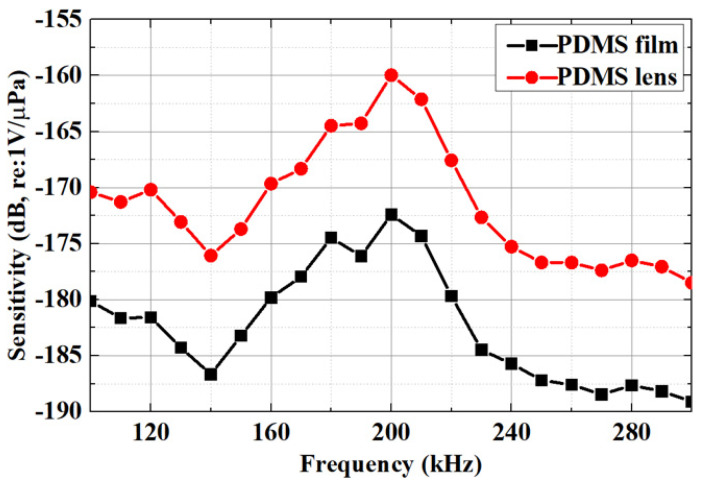
Comparison of acoustic pressure sensitivity of PMUTs with convex lenses and PMUTs without lenses. The PMUTs with convex lenses had approximately 8 dB more acoustic pressure sensitivity than the PMUTs without lenses.

A detailed comparison of the acoustic pressure sensitivity between the PMUT with a convex lens and the PMUT without a lens reveals a significant difference, as shown in Figure 7. The PMUT with a convex lens exhibits a measured acoustic pressure sensitivity exceeding −160 dB (re: 1 V/μPa) at 200 kHz. The PMUT with the convex lens exhibits approximately 10 dB higher acoustic pressure sensitivity compared to the PMUT without a lens. The measured acoustic pressure sensitivity is in good agreement with the theoretical values.

In a carefully designed directivity experiment, we employed a standard piezoelectric transducer (PZT-180 kHz) as the receiving end, positioned within a water tank. Meanwhile, a PMUT equipped with the PDMS lens/film served as the transmitting end, precisely situated below a precision graduated turntable, maintaining a 10 cm linear distance and facing the receiving end. To drive the PMUT, we utilized an AFG31000 continuous signal generator, coupled with an ATA-4315 high-voltage power amplifier, to apply a sinusoidal pulse AC signal with a frequency of 200 kHz, spanning five cycles, and an amplitude of 30 Vpp. At the receiving end, we relied on a DSOX3014G digital storage oscilloscope for precise data acquisition. Initially, we adjusted the signal to its maximum intensity in the horizontal direction. Subsequently, by rotating the precision graduated turntable, we identified the specific graduation at which the signal reached its maximum and marked it as the 0° position. Immediately following that, we rotated the turntable from −100° to 100° in 1° increments, capturing the amplitude data of the received signals sequentially. Ultimately, we normalized the collected voltage amplitude data.

**Figure 8:**
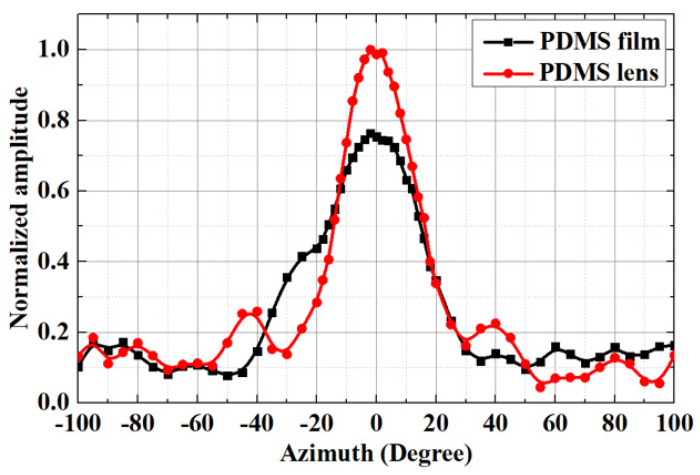
The normalized directivity at various azimuthal angles is obtained from measurements with both a convex lens and no lens for the PMUTs.

The results of directional testing revealed variations in sensitivity at different azimuthal angles for the PMUTs, both with and without the convex lens, as illustrated in Figure 8. The presence of the convex lens enhances the directivity of the PMUT, showing a more focused and directional response compared to the configuration without a lens. These findings underscore the importance of the lens in shaping and optimizing the directional sensitivity of the PMUT for specific applications.

Table 3 provides a comprehensive illustration of the performance comparison between the reported PMUT with a PDMS lens and those documented in the literature, as well as with advanced commercially available transducer. The PMUT with a PDMS lens, as investigated in this study, shows exceptional performance, characterized in particular by its comparatively high sensitivity. This promising result suggests significant potential for commercial applications.

## 6. Conclusions

This paper presents a successful exploration of performance enhancement of Piezoelectric Micromachined Ultrasonic Transducers (PMUTs) through the incorporation of PDMS acoustic lenses. The fabrication of high-performance PDMS lenses has proven to be a key factor in significantly improving the sensitivity of the PMUTs, as evidenced by the achieved sensitivity of −167.5 dB (re: 1 V/μPa). The observed enhancement in the −3 dB main lobe width within the frequency response further supports the efficacy of the PDMS lens design. Through detailed performance comparisons, it has been established that the designed PMUT in this study surpasses its counterparts documented in the literature and commercially available transducers. The promising outcomes obtained underscore the considerable potential of the designed PMUT for diverse commercial applications in the field of ultrasonic transduction.

## Figures and Tables

**Table 1:** Detailed design parameters of PMUT array.

Parameter	Value
Array length	4 mm
Array width	4 mm
Piezoelectric layer thickness	1 μm
Diaphragm characteristic size	500 μm
Electrode thickness	0.3 μm
Top Oxide layer thickness	0.2 μm
Bottom Oxide layer thickness	1 μm
Gap height	300 μm
Characteristic size	500 μm
Number of cells per array	56

**Table 2:** Lens properties.

	Property	No Lens (Water)	PDMS Film	PDMS Lens
Lens Material	$v_{l}$ (m/s)	1480	930	930
	ρ (kg/m^−3^)	$1.0\times 10^{3}$	$0.97\times 10^{3}$	$0.97\times 10^{3}$
	*Z* (MRayl)	1.48	0.9	0.9
	$\alpha_{0}$ at 6 MHz (dB/cm)	0.0022	31.0	31.0
	$\alpha_{f}$ (dB/cm/MHz)	−	7.6	7.6
Lens Geometry	Shape	−	No lens	Convex
	Radius (mm)	−	42.64	42.64
	Thickness (mm)	−	4	4
Response	Resonance frequency (kHz)	221	183	183
	Sensitivity (re: 1 V/μPa)	−163	−168	−160

**Table 3:** Comparative analysis of the performance between the developed PMUT with a PDMS lens and advanced commercially available transducers.

Hydrophone	Technology	Encapsulation	Lens	Size	Sensitivity (dB, re: 1 V/μPa)
DophinEar DE200 [8]	Piezoceramic	Polyurethane	No	cm level	−209 ± 1.5
Aquarian H2a [9]	Piezoceramic	Polyurethane	No	cm level	−180 ± 4
Brüel&Kjær 8103 [10]	Piezoceramic	Polyurethane	No	cm level	−211 ± 2
Ref. [17]	AlN	Polyurethane	No	3.5 mm × 3.5 mm	−182 ± 0.3
**This work**	ScAlN	PDMS	No	4 mm × 4 mm	**−168**
**This work**	ScAlN	PDMS	Yes	4 mm × 4 mm	**−160**

## Data Availability

The data are available upon request from the authors.
